# Good Science, Good Sense and Good Sensibilities: The Three Ss of Carol Newton

**DOI:** 10.3390/ani6110070

**Published:** 2016-11-11

**Authors:** Adrian J. Smith, Penny Hawkins

**Affiliations:** 1 Norecopa, c/o Norwegian Veterinary Institute, P.O. Box 750 Sentrum, Oslo 0106, Norway; 2 Science Group, Research Animals Department, RSPCA, Wilberforce Way, Southwater, West Sussex RH13 9RS, UK; penny.hawkins@rspca.org.uk

**Keywords:** Carol Newton, Good Science, Good Sense, Good Sensibilities, Three Ss, Three Rs

## Abstract

**Simple Summary:**

The use of animals in research is controversial and there are ongoing efforts to refine and limit their use to an absolute minimum. The Three Rs principle developed by William M. S. Russell and Rex L. Burch in the UK in the 1950s is widely used for this purpose: Replacement, Reduction and Refinement. Another useful principle which complements the Three Rs was proposed by Carol Newton in the 1970s. This principle is the Three Ss: Good Science, Good Sense and Good Sensibilities. Unlike the Three Rs, the Three Ss have not been described in detail in the literature. The purpose of this paper is to increase awareness of the Three Ss, which are a useful supplement to the Three Rs, improving animal welfare and leading to better science.

**Abstract:**

The Three Rs principle of Replacement, Reduction and Refinement developed by William M. S. Russell and Rex L. Burch in the 1950s has achieved worldwide recognition as a means of reducing the impact of science on animals and improving their welfare. However, application of the Three Rs is still far from universal, and evidence-based methods to implement the Three Rs are still lacking in many areas of laboratory animal science. The purpose of this paper is to create interest in a less well-known but equally useful principle that complements the Three Rs, which was proposed by the American biomathematician Carol M. Newton in the 1970s: the Three Ss—Good Science, Good Sense and Good Sensibilities.

Recognition of the moral imperative to consider laboratory animal welfare has a long history. In his *Principles of Investigation in Physiology*, Marshall Hall (1790–1857) proposed the replacement of animals where possible by observational studies, the necessity of clear objectives, avoidance of unnecessary repetition and the least possible infliction of suffering [1]. Then, over one hundred years later, the concept of the Three Rs (Replacement, Reduction and Refinement) was developed by William M. S. Russell and Rex L. Burch, as part of a project initiated by the Universities Federation for Animal Welfare (UFAW) and described in detail in their book, *The Principles of Humane Experimental Technique*, which was published in 1959 [2].

Despite this, the Three Rs concept was not widely embraced until the 1980s. Its profile was raised further by the establishment of a series of World Congresses on Animal Use and Alternatives, the first being held in Baltimore in 1993 [3]. This renaissance of the Three Rs led to the current global recognition of the concept and its use to underpin many national laws and codes of practice regulating animal care and use.

One significant event in the intervening period was an international symposium entitled *The Future of Animals, Cells, Models and Systems in Research, Development, Education and Testing*, organised by the U.S. Institute of Laboratory Animal Resources (ILAR) in Washington, D.C. in October 1975 [4]. The purpose of the symposium was to examine the contributions of animal experiments to human health and welfare; the use and limitations of cell, tissue and organ cultures; and the application of statistical and computer technology to biomedical research. A key issue at the symposium was whether living animals could be replaced by *in vitro* techniques and computer simulations. Carol Newton, who was then Chair of the Department of Biomathematics at the University of California in Los Angeles, was an invited speaker.

Carol Newton’s presentation was entitled *Biostatistical and Biomathematical Methods in Efficient Animal Experimentation*. She drew the attention of the participants to the challenges of biological variation and the unknown interactions between variables. She saw the role of biomathematicians as vital in understanding these processes and drew attention to the lack of “courses that include an interrelated blend of computer, statistical and modeling methods with primary emphasis on their application to real biomedical problems” [5]. Poor experimental design and inappropriate statistical analysis are still major issues in laboratory animal science [6,7].

Later in the symposium, the Canadian veterinary pathologist Harry C. Rowsell (1921–2006) spoke on *The Ethics of Biomedical Experimentation*. In the penultimate paragraph of the record of his presentation, he emphasised “*…the requirement to practice, in animal experimentation, the concept of replacement, reduction and refinement. All are consistent with the production of valid, meaningful results that, hopefully, will allow replacement of partially successful methods of disease treatment, in both man and animal, with those that are fully effective. To the three R’s we may add Carol Newton’s three S’s: good science, good sense and good sensibilities*” [8].

This is, to the best of our knowledge, the only primary reference to Carol Newton’s Three Ss in the literature. However, we believe they deserve a place alongside the Three Rs as a valuable tool to help reduce the impact of science on animals.

## 1. The Relevance of the Three Ss Concept to Laboratory Animal Science and Welfare Today 

Since, as far as we are aware, Carol Newton did not publish a description of her concept, we offer our own interpretation:

***Good Science*** is naturally the aim of all scientists. As Carol Newton pointed out, ‘experiments should be designed to reduce the effect of certain uncontrollable sources of variation, to permit effective techniques to be used in their analysis, and in general to obtain the most information with greatest certainty in the shortest time using the fewest subjects’ [5]. However, as mentioned above, there are currently very serious concerns about the quality of experimental design, statistical analysis, reporting in the literature, and the rigour of peer review within the life sciences [6,7,9,10]. All of these flaws have serious ethical implications, because poor science and reporting can lead to wasted funding, false hopes for patient groups, wastage of animals in projects of limited benefit, unnecessary suffering and, potentially, avoidable repetition of animal experiments. Current research culture is likely to underlie many of these problems (see [11]) and a prime solution would be better training and continuing education in relevant areas such as good research practice, peer reviewing and ‘laboratory leadership’ [7,11].

***Good Sense*** is an essential tool in laboratory animal science, since this is a relatively young discipline with many uncharted areas. In our opinion, good sense can help to ensure that “the Right animal is used for the Right Reason” (the Three Rs of Harry Rowsell) [12], and that *commonsense* prevails when scientific evidence is lacking. As Carol Newton said in her presentation: ‘One certainly must remain mindful of the risk that the “correct” model is not among those being considered’ [5]. In practice, when focusing on a biological system or mechanism of interest, it is essential that the researcher critically reflects on the models and approaches that have traditionally been used in the field. There is considerable debate regarding the translatability of a range of animal “models” [13,14] and researchers should challenge themselves as to *whether* animal use is valid, translatable and justified, and if so *how* animals should be used. For example, it may be possible to increase translatability and reduce adverse effects by studying biomarkers and mechanisms as opposed to creating a disease “model”. If it is deemed necessary and justifiable to create an animal model of a disease, critical reflection and review is also essential to apply the Three Rs, maximize translatability and gain the most benefit from it.

Although extrapolation from knowledge of other species, including humans, is an everyday part of planning animal research, such extrapolation must be performed with caution. For example, the great differences in metabolic rate between animals of varying size make it essential to use allometric scaling when calculating a suitable dose for novel species [15]. Besides fundamental issues such as this, common sense is essential when creating experimental protocols. In particular, translatability of animal research can be poor if drugs are administered to animals by routes which are normally irrelevant in clinical trials (e.g., by intraperitoneal injection), or if the drug is given at an inappropriate stage in the animal model—both of which still occur within preclinical trials [16].

Effective training in the biology, behaviour and welfare needs of the study species, in critical and innovative thinking, and in the scientific method helps to ensure better sense and better science. However, few researchers are currently receiving in-depth training in these subjects, either at an early stage or throughout their careers [17,18]. As the number of courses in Laboratory Animal Science increases worldwide, this should be borne in mind. These subjects are, for example, mentioned explicitly in Annex V of the EU Directive 2010/63 on the protection of animals used for scientific purposes [19].

***Good Sensibilities*** refers to the capacity to respond to, or be affected by emotions or events. Empathy for the research animal is a prerequisite for the reduction of suffering and a “life worth living” for the animals [20]. Carol Newton emphasised that experimentation on intact animals should be resorted to ‘only when necessary and by designing experiments as effectively as possible’ [5]. The Good Sensibilities aspect of the Three Ss principle is therefore also an encouragement to practice what has become known in recent years as critical anthropomorphism [21]. Assuming that interventions that would cause pain or distress to humans may also cause animals to suffer, taking the biology and behaviour of the species into account, is a good starting point for recognizing and addressing animal suffering. It will also reduce the likelihood of the animal experiencing “contingent suffering”, for example due to transport stress, inappropriate husbandry, concurrent disease and undesirable social interactions [22]. Contingent suffering has the potential to cause as much, if not more, suffering than the experimental procedures themselves, as well as the capacity to confound the science.

Although ‘sensibilities’ has largely been replaced in modern English by ‘sensitivity’ and ‘care’, it is interesting to note that a “culture of care” has become an important concept in laboratory animal science, aiming not only to improve animal welfare but also to improve conditions for animal facility staff [22,23]. More recently, researchers have been encouraged to practice a “culture of challenge” [24], asking whether established procedures are the best ones and striving to find the acceptable, rather than choosing the accepted.

It is worth noting that both Good Sense and Good Sensibilities will further Good Science, just as the Three Rs promote better science and animal welfare.

## 2. Conclusions

All of the concepts that comprise both the Three Rs and the Three Ss are essential principles of humane experimentation and should underpin the training of personnel at all career stages. We believe that this will lead to more humane, valid science for as long as animal research and testing continues.

Carol Newton stated that it was her participation on National Institutes of Health committees, and her involvement in collaborative programs during the Cold War bringing together scientists from East and West, which was her most fulfilling work [25,26]. In the discussion following her presentation in Washington [5], she was asked if she could help the proponents and opponents of animal research better understand each other. She replied: ‘*…I think the new generation of biologists…is embarking on a more quantitative orientation towards research. Almost inevitably, this will imply that they will attempt to define their terms better. When one does define one’s terms better and more exactly, one does communicate better. My feeling is that we all really have the same intent. I believe that good communication is going to be one of the things that will bring us together. Intent, though, is a human virtue, not a virtue of the computer*’.

Her concept of the Three Ss and her firm belief in dialogue between stakeholders are equally relevant today. The aim of this paper is to bring the Three Ss of Carol Newton into the limelight again, as was done with the Three Rs of Russell and Burch in the 1980s.

## Appendix

The following text, with minor modifications, is taken from a biography published by the U.S. National Library of Medicine [25,26].

### Carol M. Newton (1925–2014)

Carol M. Newton was a pioneer in the field of medical informatics for over four decades, blending the arts of medicine, mathematics and computer science. She graduated from Stanford University in 1947 and remained there to take a Ph.D. in high-energy physics. Towards the end of her doctoral work, she read papers that attracted her to new frontiers in medicine and biology which invited mathematically-supported research. She then enrolled in medical school at the University of Chicago and while still a student worked as an associate scientist at the University’s Argonne Cancer Research Hospital. She was among the first to investigate the use of analogue computers in biological sciences. She received her M.D. degree in 1960 and began a teaching career as assistant professor in both medicine and mathematical biology at the University of Chicago. In 1967 she returned to her home state, California, and worked for the rest of her career at the University of California, Los Angeles, where she was chair of the Department of Biomathematics from 1974 to 1985. Her research interests included interactive graphics software, cellular modelling related to cancer and new methods of presenting complex data sets. She was a founding fellow of the American Institute of Biological and Medical Engineering, and a fellow of the American College of Medical Informatics.
